# Vitamin E Supplementation and Cardiovascular Health: A Comprehensive Review

**DOI:** 10.7759/cureus.48142

**Published:** 2023-11-02

**Authors:** Mayank Kumar, Prasad Deshmukh, Mayank Kumar, Asmi Bhatt, Arya Harshyt Sinha, Parth Chawla

**Affiliations:** 1 Medicine, Jawaharlal Nehru Medical College, Datta Meghe Institute of Higher Education and Research, Wardha, IND; 2 Otolaryngology, Jawaharlal Nehru Medical College, Datta Meghe Institute of Higher Education and Research, Wardha, IND; 3 Community Medicine, Jawaharlal Nehru Medical College, Datta Meghe Institute of Higher Education and Research, Wardha, IND; 4 Surgery, Jawaharlal Nehru Medical College, Datta Meghe Institute of Higher Education and Research, Wardha, IND; 5 Anatomy, Jawaharlal Nehru Medical College, Datta Meghe Institute of Higher Education and Research, Wardha, IND; 6 Pathology, Jawaharlal Nehru Medical College, Datta Meghe Institute of Higher Education and Research, Wardha, IND

**Keywords:** vitamins, cvd, alpha-tocopherol, cardiovascular disease, vitamin e

## Abstract

This article conducts a thorough investigation into the potential role of vitamin E in preventing cardiovascular diseases (CVDs) in the context of shifting mortality patterns from infectious diseases to the continued prominence of CVDs in modern medicine. The primary focus is on vitamin E's antioxidant properties and its specific ability to counter lipid peroxidation, a pivotal process in the early stages of atherosclerosis, a precursor to CVDs. The research spans a wide range of methodologies, including in vitro, in vivo, clinical, and experimental studies, examining how vitamin E affects critical aspects of cardiovascular health, such as signaling pathways, gene expression, inflammation, and cholesterol metabolism. It also explores vitamin E's influence on complex processes like smooth muscle cell development, oxidative stress reduction, foam cell formation, and the stability of atherosclerotic plaques. In the context of clinical studies, the article presents findings that both support and yield inconclusive results regarding the impact of vitamin E supplementation on CVDs. It acknowledges the intricate interplay of factors such as patient selection, pathophysiological conditions, and genetic variations, all of which can significantly influence the efficacy of vitamin E. The article underscores the need for ongoing research, with a specific focus on understanding the regulatory metabolites of vitamin E and their roles in modulating cellular processes relevant to CVDs. It highlights the potential for innovative therapeutic approaches based on a deeper comprehension of vitamin E's multifaceted effects. However, it also candidly addresses the challenges of translating clinical trial findings into practical applications and emphasizes the importance of considering diverse variables to optimize therapeutic outcomes. In summary, this meticulously conducted study provides a comprehensive examination of vitamin E's potential as a preventive agent against CVDs, recognizing the complexity of the subject and the need for continued research to unlock its full potential in cardiovascular health.

## Introduction and background

In recent times, significant strides in contemporary medicine and healthcare have shifted the landscape of human mortality and morbidity away from infectious diseases. Instead, the prevailing global challenge is cardiovascular disease (CVD), which has assumed the mantle of the foremost non-communicable cause of mortality and morbidity worldwide. To illustrate the gravity of the situation, in 2023, heart disease held the unenviable distinction of being the leading global cause of mortality [1]. By 2019, a staggering 35.6 million individuals across the globe grappled with incapacitation due to CVD, translating into a societal cost of an estimated 330 million years of life [2,3]. In that same year, CVD claimed the lives of over 17 million people worldwide, and if present trajectories persist, this grim figure is projected to ascend to nearly 23 million by 2030 [4]. At the heart of the pathogenesis of CVD lies lipid peroxidation, a pivotal early event in the genesis of atherosclerosis, the precursor to CVDs [5]. Within this context, vitamin E, a fat-soluble antioxidant, emerges as a key protagonist, exerting its influence by attenuating lipid peroxidation in experimental settings [6]. Among the diverse isomers of vitamin E, alpha-tocopherol assumes a prominent role in thwarting lipid peroxidation [5]. This efficacy is grounded in alpha-tocopherol's capacity to intercept radicals, furnishing them with hydrogen atoms and thereby precluding the formation of tocopheroxyl radicals. Consequently, the tocopheroxyl radicals play a pivotal role in preventing lipid peroxidation as they engage in reactions with other radicals, ultimately leading to the generation of inactive metabolites [6].

In the realm of nutritional practices, the consumption of antioxidants, including vitamin E, selenium, carotenoids, and vitamins A and C, has gained prominence, with a substantial portion of the American population, approximately 28% to 30%, turning to antioxidant supplements [6]. Vitamin E, in particular, has garnered attention due to its anti-atherogenic and antioxidant attributes, which have been correlated with improved cardiovascular well-being in multiple research investigations, thereby resulting in a significant increase in alpha-tocopherol levels in response to vitamin E intake [7-9]. Yet the narrative is not devoid of complexity. Divergent study outcomes have surfaced; while some suggest a protective role of supplemental vitamin E against CVDs, others do not, and a few even hint at the possibility of adverse effects [7-11]. This underscores the nuanced nature of the relationship between vitamin E and CVDs. Should vitamin E indeed exert a substantial protective effect against CVDs, its integration into daily dietary regimens could hold the potential to enhance cardiovascular health on a population-wide scale. Conversely, should no substantial link between vitamin E and CVDs be established, this may prompt a reconsideration of the distribution of surplus vitamin E, thereby averting potential adverse health consequences.

It is noteworthy that alpha-tocopherol, alongside other antioxidant-rich fruits and vegetables, stands as a reliable indicator of improved cardiovascular and overall health [11]. Although other dietary antioxidants and minerals may also play contributory roles, vitamin E's potential in CVD prevention merits exploration. Consequently, an evaluation was undertaken to elucidate the connection between vitamin E and CVD, encompassing both its incidence and mortality aspects.

## Review

Methodology

**Figure 1:**
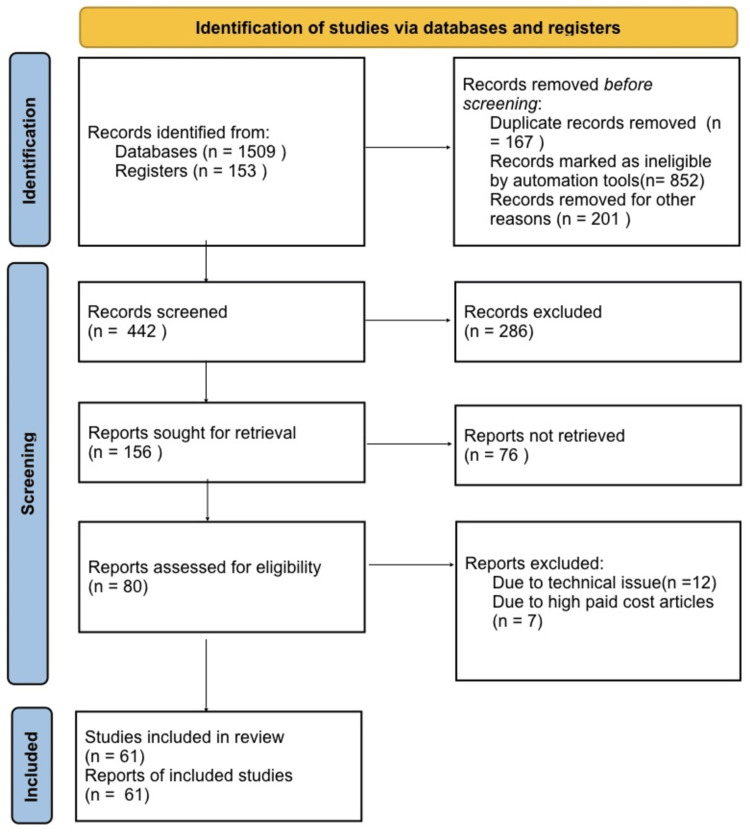
Search strategy utilized for the review. Records that were deleted for additional reasons included the language not being in English.

In conducting this narrative review, we initiated our search process by accessing databases from 'PubMed', 'Scopus', and 'Cochrane Library.' Our search encompassed key terms: 'vitamin E,' 'alpha-tocopherol,' 'cardiovascular disease,' and 'clinical investigation'. We specifically focused on English-language publications. When several reports from a single study were found in the literature, we gave priority to the articles published before 2020, or before the COVID-19 pandemic. Our inclusion criteria were designed to incorporate only review papers or studies that introduced fresh insights and findings before the pandemic. A visual representation of our search strategy can be found in Figure 1.

Discussion

Numerous investigations, spanning the domains of in vitro, in vivo, clinical, and experimental research, have harnessed tocopherol, the biologically most potent variant of vitamin E. Vitamin E, a fat-soluble vitamin, has been implicated in the modulation of diverse diseases, showcasing its multifaceted influence on biological processes, including gene expression, signal transduction, and cellular proliferation. These inquiries have unveiled the significant impact of tocopherol on various signalling pathways and gene expression, thus augmenting its potential to avert CVDs. For instance, studies involving both rat aortic artery (A7r5) and human aortic artery (HAI) cell lines have underscored that the presence of alpha-tocopherol impedes the proliferation of aortic smooth muscle cells (VSMCs) [12-14].

Alpha-tocopherol's prowess extends to its ability to curb superoxide production in human adherent monocytes by inhibiting protein kinase-C (PKC) via mechanisms such as blocking NADPH-oxidase synthesis, p47 translocation, and phosphorylation [15]. Notably, research conducted by Upston et al. revealed that tocopherol, while influential in several aspects, did not exhibit a pronounced reduction in lipid peroxidation within lesions associated with cardiovascular disease [15]. Further investigations, conducted by Azzi's group, showcased that alpha-tocopherol injections led to reduced oxidized low-density lipoprotein (oxLDL) absorption by lowering CD36 expression in HL-60 macrophages, human smooth muscle cells (SMCs), and THP-1 (human leukaemia monocytic cell) linemonocytes [16-18]. The suppression of foam cell formation, mitigation of oxidative stress, and apoptosis reduction were notable outcomes observed in the murine macrophage RAW264.7 cell line when subjected to the combined effects of high-density lipoprotein (HDL) and vitamin E [19].

These findings collectively underscore the intricate and multifaceted role of vitamin E, particularly alpha-tocopherol, in modulating various cellular processes directly relevant to CVDs, thereby underscoring its potential as a key player in the pursuit of cardiovascular health.

Vitamin E as an Antioxidant 

Animal models have served as valuable tools in the examination of vitamin E's potential in preventing atherosclerosis, a hallmark of CVD. In these studies, supplemental vitamin E, administered in the form of palm, olive, or sunflower oil, exhibited good tolerance among mice bearing aortic atherosclerotic lesions (Table 1) [20]. Notably, in these experiments, the vitamin E supplements were employed at lower concentrations, coinciding with a reduction in aortic root and arch dimensions in animals afflicted with descending thoracic aortic atherosclerosis [21]. Further insights emerged when vitamin E supplementation was observed to diminish atherosclerotic lesions in mice devoid of the low-density lipoprotein receptor (LDLR) [22]. Additionally, in a rat atherosclerosis model induced by homocysteine and cholesterol, vitamin E demonstrated a profound capacity to mitigate aortic damage, characterized by morphological indicators such as collagen accumulation and elastic fibre dissociation [23].

**Table 1:** Findings of different review articles included in this study.

Author Name	Findings
Cesare et al. [1]	Deaths from CVD jumped globally from 12.1 million in 1990 to 20.5 million in 2021.
Clarke et al. [5]	Provides insights into the role of vitamin E in health and disease.
Navab et al. [6]	Discuss the role of oxidized lipids and HDL in atherogenesis.
Colombo [7]	Provides an update on the different forms of vitamin E.
Gahche et al. [8]	Examines the increase in dietary supplement use among U.S. adults.
Steinberg et al. [9]	Discusses factors contributing to the atherogenicity of LDL.
Wright et al. [10]	Suggests an association between vitamin E and lower mortality.
Mezzetti et al. [11]	Links vitamin E and cardiovascular events in elderly individuals.
Boscoboinik et al. [12]	Investigates the effects of vitamin E on vascular smooth muscle cells.
Ozer and Azzi [14]	Explores the impact of vitamin E on atherosclerosis development.
Upston et al. [15]	Examines the role of alpha-tocopherol in the accumulation of oxidized lipids in atherosclerosis.
Ricciarelli et al. [16]	Investigates vitamin E's impact on CD36 receptor expression.
Munteanu et al. [17]	Examines the interaction between oxidized LDL and vitamin E on CD36 expression.
Huang et al. [18]	Studies vitamin E's role in reducing foam cell formation.
Su et al. [19]	Investigates the preparation and protective effects of vitamin E-containing HDL.
Ferre et al. [20]	Examines the effects of diet on lipid peroxidation and antioxidants in mice.
Thomas et al. [21]	Discusses the inhibition of atherosclerosis with co-supplementation of vitamin E and CoQ10.
Meydani et al. [22]	Addresses the effects of vitamin E on atherosclerosis and mortality.
Kirac et al. [23]	Investigates the protective role of vitamin E in a rat model.
Zani et al. [24]	Provides insights into scavenger receptors in health and disease.
Febbraio et al. [25]	Discusses the role of CD36 scavenger receptor in various processes.
Tang et al. [26]	Studies the conditional inhibition of atherosclerosis by vitamin E.
Ozer et al. [27]	Investigates the effects of vitamin E and probucol on atherosclerosis in rabbits.
Ozer et al. [28]	Explores the impact of vitamin E on CD36 receptor expression in hypercholesterolemic rabbits.
Yazgan et al. [29]	Links CD36 expression to the onset of atherosclerosis in peripheral blood mononuclear cells.
Sozen et al. [42]	Examines changes in oxysterol and scavenger receptor levels in heart tissue due to a high cholesterol diet.
Qureshi et al. [43]	Studies the effects of tocotrienol-rich fraction on serum cholesterol.
Packer et al. [44]	Discusses the molecular aspects of alpha-tocotrienol's antioxidant action.
Azzi et al. [45]	Explores non-antioxidant functions of alpha-tocopherol.
Sen et al. [46]	Discusses the potential of tocotrienols in cardiovascular disease.
Ramanathan et al. [47]	Highlights tocotrienol's cardioprotective role.
Azzi et al. [48]	Discusses the historical rise, fall, and renewed interest in vitamin E.
Watkins et al. [49]	Examines the use of multivitamins and its association with mortality.
Muntwyler et al. [50]	Investigates vitamin supplement use and cardiovascular mortality in a low-risk population.
Stephens et al. [51]	Discusses a randomized trial of vitamin E in patients with coronary disease.
Boaz et al. [52]	Discusses a randomized trial of antioxidants in cardiovascular disease prevention.
deOliveira et al. [53]	Examines the associations between dietary intakes and cardiovascular disease risk.
Devaraj and Traber [54]	Explores the role of gamma-tocopherol in contrast to alpha-tocopherol.
Ohrvall et al. [55]	Investigates serum levels of gamma-tocopherol in coronary heart disease patients.
Devaraj et al. [56]	Examines the effects of gamma-tocopherol supplementation.
Vucinic et al. [57]	Studies the effects of gamma-tocopherol supplementation on exercise-induced effects.
Stonehouse et al. [58]	Investigates the effects of palm-tocotrienol and palm-carotenes on vascular function and cardiovascular disease risk.
Eshak et al. [59]	Explores the association between dietary fat-soluble vitamins and mortality from heart failure.
Wang et al. [60]	Investigates the impact of vitamin C and vitamin D on mood and distress in hospitalized patients.
Loffredo et al. [61]	Suggests an association between vitamin E supplementation and reduced myocardial infarction.

Within the context of early atherosclerosis, the presence of macrophage foam cells is indicative. These cells have been found to harbour scavenger receptors, a subtype of membrane-bound receptors which avidly bind ligands such as oxidized phospholipids/lipoproteins and modified lipid particles [24]. CD36, a prominent scavenger receptor within macrophage membranes, has been identified in numerous cell types, including monocytes/macrophages, endothelial cells, and SMCs, and is recognized as a pivotal player in the context of CVD. Importantly, CD36 plays a critical role in foam cell formation during the atherogenic process [25]. Significantly, treatment with alpha-tocopherol in ApoE-deficient (ApoE) mice was found to concurrently reduce CD36 mRNA expression while enhancing the expression of transcription factors like PPAR (peroxisome proliferator-activated receptors), LXR (liver X receptor), and catenin [26]. These findings illuminate the intricate interplay between vitamin E, cellular mechanisms, and atherosclerosis, offering valuable insights into the potential therapeutic avenues that may emerge from such research endeavours.

Antioxidant Role of Vitamin E in Preventing CVD and CVD Mortality

Hypercholesterolemia significantly escalates the risk of atherosclerosis and ischemic diseases like myocardial infarction and cerebral infarction. Within our laboratory's high-cholesterol diet-fed rabbit model, we have unearthed compelling evidence showcasing the preventive potential of vitamin E in the context of atherosclerosis development. Specifically, alpha-tocopherol, a form of vitamin E, has been shown in previous in vivo studies to curtail the progression of cholesterol-induced atherosclerotic plaques by diminishing protein kinase C (PKC) activity [27]. Our research team has further elucidated that alpha-tocopherol effectively suppresses the production of CD36 mRNA, thereby mitigating the development of cholesterol-mediated atherosclerotic lesions [28]. This phenomenon has been substantiated in our rabbit model, where vitamin E was found to reduce CD36 mRNA production in aortic tissue and peripheral blood mononuclear cells [29]. Atherosclerosis progression is closely associated with cholesterol transport in the form of oxLDL, which stimulates a signalling cascade involving matrix metalloproteinase (MMP), c-jun N-terminal kinase-1 (JNK1), and mitogen-activated protein kinase (MAPK), thereby promoting inflammation and monocyte infiltration. Our investigations have shed light on alpha-tocopherol's ability to dampen JNK1-mediated c-jun phosphorylation, proteasome activity, and MMP-9 synthesis in the hypercholesterolemic atherosclerotic process [30].

Moreover, in a hypercholesterolemic rabbit model, vitamin E exhibited a decelerating effect on atherosclerosis progression by elevating the levels of PPAR and nuclear factor erythroid 2-related factor 2 (Nrf2), consequently enhancing ATP-binding cassette transporter A1 (ABCA1) and glutathione S-transferase (GST) while diminishing MMP-1 [31]. In the realm of inflammation, vitamin E's anti-inflammatory properties have been a focus of research, with three key mechanisms identified: suppression of nuclear factor-kappa B (NF-κB), reduction in PKC activity, and decreased synthesis of adhesion molecules, including vascular cell adhesion molecule-1 (VCAM-1), intercellular adhesion molecule-1 (ICAM-1), and E-selectin [32-35]. The presence of alpha-tocopherol has been correlated with reduced production of pro-inflammatory cytokines such as interleukin-1 (IL-1), IL-6, tumour necrosis factor (TNF), interferon (IFN), and IL-8 [36].

Collectively, these preliminary findings point to vitamin E's capacity to modulate gene expression and enzyme activity associated with inflammation, oxLDL uptake, and foam cell formation, thereby impacting the initiation of atherosclerosis at both cellular and animal levels. In the broader context of cardiovascular diseases (CVDs), encompassing ischemic heart disease and heart failure, a range of in vitro and in vivo models have been instrumental in research endeavors. Oral administration of L-arginine, docosahexaenoic acid, eicosapentaenoic acid, vitamin E, and vitamin C has demonstrated the potential to mitigate CVD risk factors in C57Bl/6 mice [37]. Notably, vitamin E has shown promise in preventing acute myocardial infarction induced by left anterior descending coronary artery blockage, potentially reducing mortality [38].

Heart failure, distinguished by diminished metabolic energy reserves and the activation of diverse molecular pathways leading to ventricular hypertrophy, inflammation, fibrosis, angiogenesis, and apoptosis, presents distinct challenges within the realm of CVDs [39]. Vitamin E has been shown to support cardiomyocyte health by mitigating apoptotic activity [40]. Furthermore, in a rat model of streptozotocin-induced diabetic heart failure, vitamin E supplementation offered protection when administered over an eight-week period [41,42]. Beyond vitamin E, compounds such as tocotrienols have demonstrated their potential in reducing CVD incidence by lowering blood cholesterol levels and triglycerides, two pivotal CVD risk factors [43]. Tocotrienols influence cholesterol metabolism by mitigating LDL oxidation and regulating the expression of 3-hydroxy-3-methylglutaryl-coenzyme A reductase, a key enzyme in cholesterol synthesis [44]. Additionally, our findings, in alignment with animal studies, suggest that tocotrienols may hold promise in averting atherosclerosis [45,46]. Their ability to activate proteasomes and enhance myocardial health has positioned tocotrienols as a recognized cardioprotective agent [47].

Investigations

Vitamin E, as substantiated by numerous in vitro and in vivo studies, plays a pivotal role in the regulation of signal transduction, inflammation, cholesterol metabolism, and the stability of atherosclerotic plaques. Importantly, long-term clinical studies have illuminated vitamin E's protective potential against the onset of cardiovascular diseases (CVD) [48]. Specifically, vitamin E has emerged as a guardian against select cardiovascular ailments, notably coronary heart disease. A notable reduction in the incidence of ischemic heart disease, ranging from 10% to 14%, was observed in women who supplemented their diets with vitamin C, E, and/or vitamin A, either individually or in conjunction with multivitamins [49]. Further studies have revealed that the prolonged use of vitamin E supplements, exceeding four years, resulted in a remarkable 59% reduction in mortality from coronary disease [50]. In individuals afflicted with coronary atherosclerosis, the administration of tocopherol at doses ranging from 400 to 800 mg/dL yielded a decreased incidence of myocardial infarction, as demonstrated by the Cambridge Heart Antioxidant Study [51]. An investigation into the secondary prevention of cardiovascular disease with antioxidants in patients found that the administration of alpha-tocopherol (800 mg/dL) significantly reduced the composite endpoint of myocardial infarction (fatal and non-fatal), ischemic stroke, and chronic kidney disease in patients with end-stage renal disease [52].

The Multi-Ethnic Study of Atherosclerosis also affirmed the beneficial effects of adequate vitamin E consumption in mitigating risk factors for cardiovascular conditions [53]. Numerous clinical studies have explored the inverse relationship between alpha-tocopherol and coronary heart disease, either in isolation or in conjunction with other analogues [54,55]. In patients with metabolic syndrome, treatment with alpha-tocopherol, alone or in combination with other antioxidants, led to reduced markers of oxidative stress and conferred protection against exercise-induced increases in coagulation and platelet aggregation [56, 57]. However, it is important to note that not all studies have yielded uniformly positive results. For instance, a randomised controlled experiment investigating the benefits of palm tocotrienols failed to reveal significant reductions in CVD risk factors or improvements in vascular function [58].

Furthermore, the relationship between vitamin E supplementation and dietary intake has been explored. Japanese women who consumed higher amounts of fat-soluble vitamins, including vitamin E, in their diets exhibited a reduced risk of heart failure-related mortality, as reported by Ehab et al. [59]. This association, however, did not hold true for men. In another study, the extended use of vitamin E supplements, surpassing two years, was associated with a notable 41% reduction in the risk of developing cardiovascular disease (CVD) [60]. On the interventional front, Loffredo et al. underscored that vitamin E therapy in isolation reduces the incidence of myocardial infarction, but its effectiveness diminishes when administered alongside other antioxidants [61].

These collective findings underscore the multifaceted role of vitamin E in cardiovascular health and highlight the potential benefits of its supplementation in select contexts, while acknowledging that further research and nuanced considerations may be necessary to fully elucidate its impact on diverse populations and clinical scenarios. The findings of the different studies included in this review are shown in Table 1.

## Conclusions

As of 2023, CVD continues to stand as the leading cause of both mortality and morbidity, accounting for a significant 32% of all deaths. This ongoing concern is further exacerbated by the escalating prevalence of obesity and diabetes, both of which are substantial risk factors on the rise worldwide. Extensive research has consistently shown a strong link between reduced vitamin E levels and various diseases, with a particular emphasis on cardiovascular disease. Pioneering studies in animal and cell culture models have provided valuable insights into the molecular pathways influenced by vitamin E and its metabolites across various disease contexts. While initial promise emerged from in vitro and in vivo research, subsequent human investigations have yielded mixed findings regarding the cardiovascular benefits of vitamin E. Nevertheless, the evolving landscape of the medical sciences has enriched our understanding of vitamin E. Of notable importance is the exploration of phosphorylated and catabolized products, including both long- and short-chain metabolites, and their potential role in regulating cellular processes relevant to CVD. Ongoing research into this category of regulatory metabolites, involving comprehensive studies at both cellular and clinical levels, holds promise for filling crucial knowledge gaps and providing insights into preventing cardiovascular disease.

Challenges still persist in translating clinical trial outcomes into meaningful practical applications, but the identification of key players within the cellular and molecular systems influenced by vitamin E offers promise for novel therapeutic options in combating cardiovascular disease. Simultaneously, a thorough grasp of metabolomics for distinguishing between various vitamin E compounds remains crucial. As emphasized in this review, factors such as patient selection, pathophysiological conditions, intervention timing, and gene polymorphisms can significantly affect vitamin E's effectiveness against CVD. Therefore, comprehensive investigations into dietary and supplemental levels, considering these factors, are essential to optimizing the therapeutic potential of various medical interventions. It is essential to stay updated with the latest available data and ongoing research to continually refine our understanding of the role of vitamin E in combating cardiovascular disease.
